# Recent Advances of Tumor Microenvironment-Responsive Nanomedicines-Energized Combined Phototherapy of Cancers

**DOI:** 10.3390/pharmaceutics15102480

**Published:** 2023-10-17

**Authors:** Kehan Liu, Yao Yao, Shujuan Xue, Mengyao Zhang, Dazhao Li, Tao Xu, Feng Zhi, Yang Liu, Dawei Ding

**Affiliations:** 1 College of Pharmaceutical Sciences, Soochow University, Suzhou 215123, China; 20224226059@stu.suda.edu.cn (K.L.); 20214226033@stu.suda.edu.cn (S.X.); 20214226050@stu.suda.edu.cn (M.Z.); 20206150004@stu.suda.edu.cn (T.X.); 2 Department of Gerontology, The Affiliated Suqian Hospital of Xuzhou Medical University, Suqian 223800, China; 20224133014@stu.suda.edu.cn; 3 Department of Neurosurgery, The First People’s Hospital of Changzhou, Changzhou 213003, China; 20204250141@stu.suda.edu.cn (D.L.); danielzhif@suda.edu.cn (F.Z.); 4 Clinical Medical Research Center, The Third Affiliated Hospital of Soochow University, Changzhou 213003, China; 5 School of Pharmacy & Biomolecular Sciences, Royal College of Surgeons in Ireland (RCSI), D02 NY74 Dublin, Ireland

**Keywords:** combination therapy, tumor microenvironment, phototherapy, chemotherapy, controlled drug release, hypoxia relief

## Abstract

Photodynamic therapy (PDT) has emerged as a powerful tumor treatment tool due to its advantages including minimal invasiveness, high selectivity and thus dampened side effects. On the other side, the efficacy of PDT is severely frustrated by the limited oxygen level in tumors, thus promoting its combination with other therapies, particularly photothermal therapy (PTT) for bolstered tumor treatment outcomes. Meanwhile, nanomedicines that could respond to various stimuli in the tumor microenvironment (TME) provide tremendous benefits for combined phototherapy with efficient hypoxia relief, tailorable drug release and activation, improved cellular uptake and intratumoral penetration of nanocarriers, etc. In this review, we will introduce the merits of combining PTT with PDT, summarize the recent important progress of combined phototherapies and their combinations with the dominant tumor treatment regimen, chemotherapy based on smart nanomedicines sensitive to various TME stimuli with a focus on their sophisticated designs, and discuss the challenges and future developments of nanomedicine-mediated combined phototherapies.

## 1. Introduction

In recent decades, photodynamic therapy (PDT) has attracted constantly increasing attention for the treatment of malignant tumors owing to the apparent merits including low toxicity, minimal invasiveness, and high selectivity by modulating a couple of parameters (e.g., power density, exposure location and duration) [1,2,3,4]. In the process of PDT, the light of a specific wavelength is employed to excite the photosensitizer (PS), which reacts with surrounding molecules to generate free radicals through direct electron transfer (Type I) for the production of reactive oxygen species (ROS) (e.g., hydroxyl radicals, superoxide and hydrogen peroxide), or reacts with the surrounding molecular oxygen (^3^O_2_) to produce highly reactive singlet oxygen (^1^O_2_)(Type II) [5,6,7]. The resultant ROS instantly induces oxidative stress and consequently cell apoptosis and necrosis for tumor ablation [7,8]. However, the efficacy and efficiency of PDT are restrained by a few detrimental limitations. Firstly, the extraordinary proliferation of tumor cells and premature tumor blood vessels due to rapid tumor growth leads to a hypoxic tumor microenvironment (TME) [9,10,11,12], which severely frustrates the ROS production of PDT, particularly for the more frequently applied Type II PDT, whereas oxygen is a key element in ^1^O_2_ generation [13]. Unfortunately, the vascular damage caused by PDT diminishes the blood and oxygen supply, which exacerbates the tumor hypoxia [7]. The second challenge comes from ROS consumption by the tumor cells. Enriched intracellular anti-oxidants including elevated levels of glutathione (GSH) can scavenge ROS produced by PSs, thus abating the PDT efficacy [14,15]. To this end, PDT has been widely combined with other therapies, such as chemotherapy, radiotherapy, photothermal therapy (PTT) and immunotherapy to benefit from their different mechanisms of eradicating tumor cells for enhanced effectiveness [5,16,17,18,19].

Among the above-mentioned therapies, PTT is a treatment strategy predicated on materials with high photothermal conversion efficiency to convert light energy into heat energy for cell ablation [20,21]. These materials absorb energy from photons of a specific wavelength and undergo a transition from their ground singlet state to an excited singlet state, whereas the electronic excitation energy then experiences vibrational relaxation, a non-radiative decay, and a return to the ground state mediated by collisions between the excited photothermal agents and their surrounding molecules. In consequence, increased kinetic energy gives rise to the heating of the surrounding microenvironment [3]. Cells behave differently depending on the temperature aroused by the photothermal agents. A minor increase to 41 °C initiates a heat-shock response that in turn elicits a series of rapid changes in gene-expression patterns (e.g., the generation of heat-shock proteins) to alleviate the effects of the initial thermal damage [22], while 42 °C leads to irreversible tissue damage, and the heating of tissues to a temperature between 42 and 46 °C for 10 min induces cell necrosis [23]. Cells rapidly die at 46–52 °C due to microvascular thrombosis and ischemia, and a temperature beyond 60 °C usually causes instantaneous cell death owing to protein denaturation and plasma membrane destruction [24].

**Figure 1:**
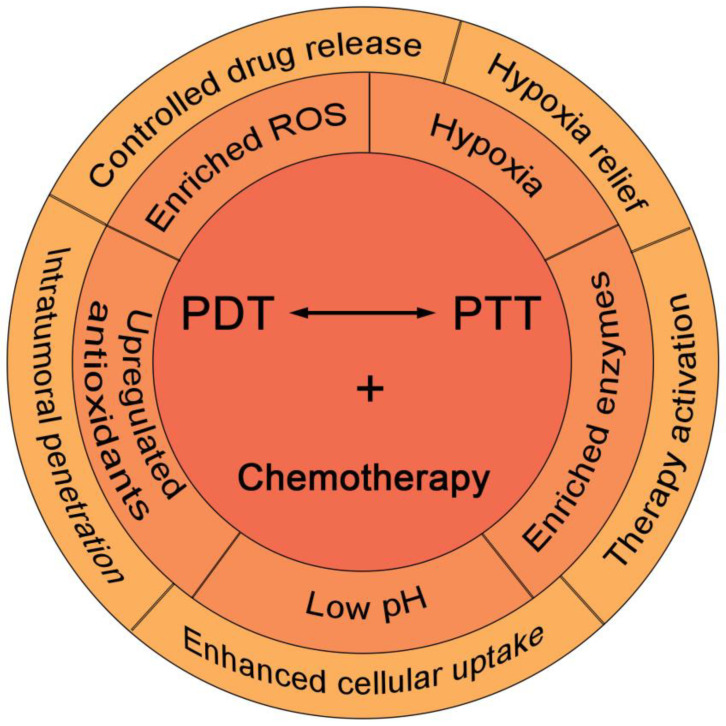
Schematic illustration showing TME-responsive nanomedicines for combined phototherapy and its combination with chemotherapy with additional benefits in drug delivery.

Owing to their dual-functionality as a PS besides photothermal agents, some materials particularly NIR dyes provide a scientific rationale and feasibility to combine PTT with PDT in tumor treatment [13,24]. Inspired by this, many sole photothermal materials (e.g., precious metal material, transition metal sulfur oxides, carbon material and upconversion nanomaterial) and PSs (e.g., phthalein cyanogen, porphyrins and other dye molecules) have also been widely used in photothermal-photodynamic synergistic treatment [24,25,26,27]. In synergistic phototherapy, the hyperthermia induced by PTT could speed up intratumoral blood flow, somehow promoting O_2_ supply for slightly mitigated tumor hypoxia and eventually leading to an increase in PDT efficacy [16]. In addition, the mild high temperature can increase the concentration of the photosensitizers in the cell by improving the permeability of the membrane, thereby increasing the accumulation of the photosensitizer-loaded nanoparticles in the tumor [28]. In this way, such treatment can target tumor cells that cannot be completely eradicated by PTT or PDT alone, and even suppress tumor recurrence and metastasis [29]. On the other side, synergistic phototherapy still suffers from a few drawbacks, namely the detrimental dependence on oxygen and limitation of tumor hypoxia, lack of control on drug release, as well as inadequate cellular uptake and intratumoral penetration of nanomedicines. To this end, stimuli-responsive nanocarriers, particularly those sensitive to TME features of tumor tissues including enriched ROS (particularly H_2_O_2_) and lower pH, are being extensively explored in combined PDT and PTT to tackle these problems [30,31,32,33]. In this review, we will summarize the recent progress of synergistic phototherapies based on smart nanomedicines sensitive to various TME stimuli (Figure 1), while the challenge and future developments of nanomedicine-mediated phototherapies will also be discussed.

## 2. Enriched H_2_O_2_-Powered Phototherapy

Tumor cells are featured by bolstered levels of ROS which are mainly produced by mitochondria metabolism [34,35]. As the dominant and most representative ROS in TME, H_2_O_2_ originates from the reaction of superoxide catalyzed by the excessively produced superoxide dismutase (SOD) [36]. Elevated levels of ROS facilitate the development, invasion and metastasis of tumors, while highly excessive ROS display cytotoxicity that leads to the apoptosis and necrosis of malignant cells due to the damage of cellular lipids, protein, and DNA [13,37]. In order to avoid the unlimited increase of ROS levels and to maintain the redox balance, tumor cells usually upregulate intracellular antioxidants, particularly elevated glutathione (GSH) levels to combat the ROS [13]. On the other side, both enriched ROS and GSH could be leveraged for improved tumor phototherapy. For instance, the enriched H_2_O_2_ in TME could benefit the alleviation of tumor hypoxia through their in situ decomposition and generation of O_2_ assisted by various catalytic substances, which could improve the efficacy of synergistic phototherapy [38,39] (Table 1). In addition, elevated GSH could also be extensively utilized in TME-responsive drug delivery to tumors for triggered drug release [17].

### 2.1. Hypoxia-Relieved Phototherapy via Enzyme-Catalyzed H_2_O_2_ Decomposition

**Figure 2:**
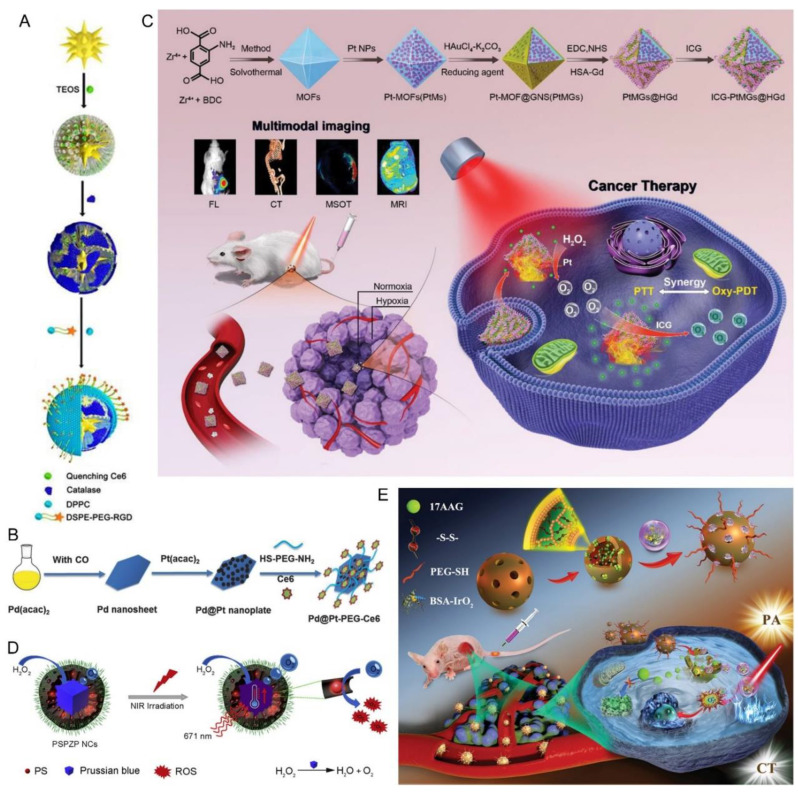
Oxygen-replenished synergistic phototherapy via catalase, and metallic nanocatalysts with/- the co-loading of PS. (**A**) Schematic illustration of the preparation process of the Au@mSiO_2_/Ce6@Catalase@DSPE-PEG-RGD probe and the tumor-targeted imaging and therapy. Adapted with the open access figure from ref. [41]. (**B**) Schematic illustration of preparing Pd@Pt-PEG-Ce6. Adapted with permission from ref. [42]. (**C**) Schematic illustration of the ICG-PtMGs@HGd nanocarriers as H_2_O_2_-driven oxygenator for multimodal imaging-guided enhanced synergistic PDT and PTT treatments in solid tumors. Adapted with permission from ref. [45]. (**D**) Schematic illustration of the photo-enhanced tumor via the PB@SiO_2_-PEG nanocatalysts loaded with ZnPc (PSPZP NCs). Adapted with permission from ref. [46]. (**E**) Schematic illustration of AHBIP nanoplatform for CT/PA imaging-guided dually-enhanced phototherapy of tumors. Adapted with permission from ref. [50].

Catalase (CAT) is the most used enzyme for the catalysis of in situ O_2_ generation for combined phototherapy [10]. In an earlier report, CAT was linked to the core-shell gold nanorods (AuNRs) modified with phenyl mesoporous silica (APMs) for the photothermal effect resulting from the longitudinal surface plasmon resonance (SPR) of AuNRs and their catalysis activity to convert the endogenous H_2_O_2_ to oxygen [40]. The cRGD-modified nanocomposites were co-loaded with methylene blue (MB) as the PS and Eu^3+^-based salt which could activate the MB acceptor via luminescence resonance energy transfer (LRET) effect, converting a deep-penetrating NIR light into a visible light to excite the later. Consequently, the nanoplatform provided synergistic PDT and PTT against hypoxic tumor cells, simultaneously solving the critical shortcoming, tumor hypoxia of PDT. However, this design did not demonstrate the efficacy of synergistic phototherapy in vivo. Later on, Zhang and the colleagues developed a novel integrated theranostic nanoplatform for controlled PS release based on gold nanostar (GNS) core and mesoporous silicon shell loaded with chlorin e6 (Ce6) and modified with CAT and the targeting moiety c(RGDyK) (named as Au@mSiO_2_/Ce6@Catalase@DSPE-PEG-RGD, ASCE-R) [41] (Figure 2A). As a result of its atomic number and X-ray attenuation coefficient, GNS allowed computed tomography (CT) imaging. More importantly, GNS “switched off” the PDT of Ce6 due to the proximity between the two, and reduced the side effects of Ce6 by preventing premature leakage. On the other side, Ce6 release was triggered when PTT was implemented by laser irradiation, enabling the generation of ROS for PDT. At the same time, CAT converted enriched H_2_O_2_ to oxygen, which solved the problem of tumor hypoxic resistance and boosted the efficacy of synergistic phototherapy.

### 2.2. Hypoxia-Relieved Phototherapy via Metallic Nanomaterial-Catalyzed H_2_O_2_ Decomposition

In addition to CAT, metallic nanomaterials with catalase-like activity represent another major category of catalytic substrates for the relief of tumor hypoxia. In particular, MnO_2_ has gained the most attention. Moreover, as a result of the decomposition tendency of MnO_2_ under the condition of lower pH and enriched H_2_O_2_ (MnO_2_ + H_2_O_2_ + 2H^+^ → Mn^2+^ + 2H_2_O + O_2_) in the TME [52], MnO_2_-based nanocarriers are usually harnessed for dual-stimuli responsiveness and their applications will be discussed in the following section.

Besides MnO_2_, Pt-based nanomaterials are also frequently involved in combination with other drugs for synergistic phototherapy due to their significant ability to catalyze H_2_O_2_ decomposition and photothermal conversion capability at the same time. Zheng’s group reported a PS-Pd@Pt nanosystem (Pd@Pt-PEG-Ce6) for highly efficient PDT and moderate PTT [42]. The catalase-like “nanoenzyme” Pd@Pt nanoplates were composed of 2D palladium nanosheets, Pt NPs and the photosensitizer Ce6 linked by mercaptoaminopolyglycol (SH-PEG-NH_2_) (Figure 2B). In addition to producing long-lasting O_2_ with intratumoral H_2_O_2_ in situ which assisted in enhancing the PDT efficacy in hypoxic tumors together with the moderate photothermal effect resulting from Pd@Pt nanoplates, covalently connected Ce6 provided a good imaging and tracing tool for the visual examination of tissue distribution as well as guiding the PDT treatment of Pd@Pt-PEG-Ce6. Bearing a similar idea in mind, Chen and colleagues developed porous Pt NPs as both a “nanozyme” and a photothermal agent to covalently load Ce6 for PDT and PTT [43]. The high photothermal conversion efficiency (52.6%) in response to 1064 nm laser irradiation significantly suppressed the tumor growth and recurrence together with oxygen-evolving PDT in the U14 tumor-bearing mice. Recently, Chen and his team coated an ultra-thin Pt shell on Pd nanocubes (Pd@Pt) by a simple liquid phase method [44]. The deposition of Pt shell on Pd nanocubes not only enhanced the catalase-like activity and durability of the nanocomposites by electron coupling and plasmon effect but also strengthened the local electric field, which greatly improved the photothermal conversion efficiency. In addition, the Pt shell sensitized and formed singlet oxygen (^1^O_2_) due to light-mediated plasmon-induced excitation, thereby achieving bolstered phototherapy.

Pursuing a multi-purpose ambition, You and co-workers designed a continuous O_2_ self-replenishing nanoplatform, Pt NP-decorated gold NPs with metal-organic frameworks (MOFs) as the inner template for multimodal imaging-guided synergistic phototherapy [45] (Figure 2C). The stable crystalline porous structure of MOFs enabled the loading of a large amount of Pt as “nanozymes”, while the porous gold nanoshells were fabricated onto them by a rapid and facile one-step method for PTT due to the SPR effect. The resultant nanocarriers were further modified with HSA-Gd hybrids (HGd) (PtMG@HGd) for MR imaging [53], and for the biocompatibility, stability, and tumor targeting ability (passive and active) of HSA [11] before the loading of indocyanine green (ICG) as the PS. On one side, ICG-PtMG@HGd nanocomposites enabled simultaneous FL/MSOT/CT/MR quadruple-modal imaging that provided more accurate tumor information from the optical, electronic, and magnetic perspectives to guide the therapy. On the other side, they also achieved persistent modulation of the hypoxia TME by Pt-mediated self-supply of oxygen, which improved the antitumor effects in terms of suppression on both the primary and metastatic tumors by synergistic phototherapy.

Besides Pt, other metals including Fe, ruthenium (Ru), iridium (Ir)and rhodium (Rh) have also been developed into nanomaterials for collaborative phototherapy, which are able to catalyze the decomposition of H_2_O_2_ and simultaneously induce photothermal conversion or enhance photodynamic therapy. For instance, Wang and colleagues designed safe and versatile nanocatalysts with Fe-based Prussian blue (PB) [46]. In detail, PB was coated with mesoporous silica to enable the loading of zinc phthalocyanine (ZnPc) as the PS, while the NP surface was modified by PEG chains (PB@SiO_2_-PEG) (Figure 2D) for enhanced aqueous stability, excellent biocompatibility, and long blood circulation time. Besides the role of catalase, the inner PB also served as a photothermal agent to trigger the increase of local temperature which additionally promoted the oxygen supply. Meanwhile, ZnPc could immediately transform the oxygen to cytotoxic ROS under the same irradiation. Consequently, they realized the photothermally controlled improvement of tumor hypoxia for enhanced cancer phototherapy. Similarly, Ruan et al. designed iron-manganese layered double hydroxide nanosheets to load methylene blue (FeMn-LDH/MB) [54]. The O_2_-enhanced PDT via the catalytic decomposition of H_2_O_2_ by the Fe/Mn composite and its dual-functional photothermal effect achieved the almost complete eradication of tumors in vivo.

Beyond the Fe-based nanomaterials, Xu et al. designed RuO_2_@BSA@IR-808-Br_2_ (RBIR) which simplified the dual-wavelength activation of PDT and PTT [47]. Under NIR irradiation, RuO_2_@BSA (RB) not only increased the local temperature as a photothermal agent but also accelerated the oxygen supply for PDT. In order to prevent the leakage of heavy metal ions and to simplify the synthesis/preparation process, Wang and his team developed a new bimetallic and biphasic rhodium (Rh)-based core-shell nanosystem loaded with ICG (Au@Rh-ICG) for PTT-boosted PDT (Au@Rh-ICG-CM) [48], whereas the porous Au@Rh NPs exhibited catalase-like activity for oxygen generation. Interestingly, a coating with tumor cell membrane (CM) enhanced the biocompatibility, tumor targeting capability and the retention of ICG before premature leakage. Cao and his team prepared a therapeutic diagnostic Ce6-Rh@MPDA (CRM) NP to achieve relief of tumor hypoxia and photothermal enhanced PDT [49]. Rh NPs could catalyze the production of O_2_ from tumor-enriched H_2_O_2_, where the mesoporous structure of mesopore polydopamine (MPDA) can further enhance catalytic activity by providing sufficient contact between the catalytic active site and the reactants. In addition, the hyperthermia induced by the combined photothermal properties of both MPDA and Rh NPs under laser irradiation not only conducted PTT but also accelerated the catalytic reaction. This study represents an example of the development of a biocompatible nanoplatform that efficiently modulate the hypoxic TME to obtain desired therapeutic performance via elevating metal-catalytic activity.

Different from the above examples that relied on metallic nanomaterials, Zhu’s team reported a hollow mesoporous organosilica nanoparticles (HMONs)-based nanoplatform for dully-improved PDT and low-temperature PTT without any PS [50]. In this study, disulfide bond-modified HMONs were used to internally load 17AAG, a typical heat shock protein 90 (HSP 90) inhibitor in the core, while BSA-IrO_2_ was attached on the surface via disulfide bonds to prevent the premature release of inner cargo before the decoration with PEG (17AAG@HMONs-BSA-IrO_2_-PEG, AHBIP) (Figure 2E). Once accumulated in the tumor, the BSA-IrO_2_ gatekeeper would be eliminated due to the cleavage of disulfide bonds via enriched GSH in the TME, which subsequently provoked the release of 17AAG. More interestingly, BSA-IrO_2_ simultaneously exhibited three features including strong NIR absorbance and photothermal conversion for PTT, photocatalysis activity for novel PDT via the generation of superoxide anions by laser irradiation, and catalase-like activity to produce O_2_ for PDT improvement. Meanwhile, the stimuli-responsive release of 17AAG specifically inhibited HSP90, which frustrated the thermoresistance of tumor cells and thus enhanced the efficacy of PTT at a relatively low temperature (≈41 °C). Consequently, the dully-enhanced phototherapy via both tumor oxygenation and HSP inhibition endowed AHBIP with outstanding therapeutic outcomes. In another more recent study, a novel nanozyme-based IrO_2_@MSN@PDA-BSA (Ce6) nanoplatform for tumor PTT and PDT was synthesized [51]. The polydopamine (PDA) coating and IrO_2_ NPs of the intelligent nanoplatform exhibited a high photothermal conversion efficiency of 29.8% under NIR irradiation, enabling the ablation of solid tumors by hyperthermia. In addition, under 660nm NIR laser irradiation, Ce6 produced abundant ROS enhanced by oxygen production catalyzed via IrO_2_ nanoparticles, which collectively eradicated the tumors.

In summary, enriched H_2_O_2_ in the TME has been extensively utilized in the synergistic phototherapy of hypoxic tumors for the improvement of PDT via oxygen supply catalyzed by enzymes and metallic nanomaterials, as well as via the enhancement of blood flow in tumors by PTT. In most cases, the metallic nanomaterials could also perform as the photothermal agents for PTT by themselves, thus omitting the usage of photothermal agents.

## 3. pH-Responsive Phototherapy

The TME is weakly acidic, and its pH value (around 6.5–7.0) is lower than that of normal tissues and blood (around 7.4), due to that they can make use of glucose to produce energy through glycolysis rather than aerobic respiration, which enhance the acidity via the massive generation of lactic acid [32]. Given this, many intelligent multifunctional nanoplatforms that are able to respond to the weakly acidic TME have been developed for combined phototherapy (Table 2). They have been specifically designed for triggered drug release and the resultant reduced side effects, enhanced cellular uptake as well as therapy activation as illustrated below.

### 3.1. pH-Responsive Drug Release

**Figure 3:**
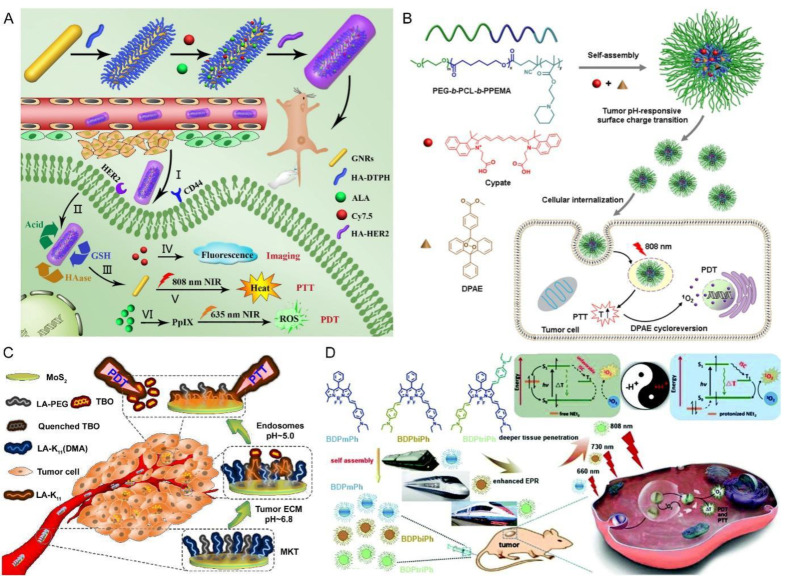
pH-responsive nanocarriers for synergistic phototherapy with pH-sensitive drug release, charge reversal- enhanced cellular uptake, and pH-triggered activation of therapy. (**A**) Schematic illustration of the preparation of GNR-HA^−ALA/Cy7.5^-HER2 with triple-responsive drug release and its application for HER2/CD44 dual-targeted and fluorescence imaging-guided combined PDT/PTT of breast cancer. (I) GNR-HA^−ALA/Cy7.5^-HER2 accumulates in tumor owing to the EPR effect. (II) GNR-HA^−ALA/Cy7.5^-HER2 is recognized by CD-44 and HER2 receptors and taken up by tumor cells. (III) The release of ALA is triggered by acidic intracellular microenvironment. HA is degraded by GSH and HAase. The Cy7.5, GNRs and ALA are harnessed for fluorescence imaging (IV), PTT(V) and PDT (VI) of HER2-positive breast cancer, respectively. Adapted with permission from ref. [56]. (**B**) Schematic illustration of C/O@N-Micelle with pH-responsive enhanced cellular uptake and oxygen-independent photothermally triggered PTT/PDT under NIR irradiation. Adapted with permission from ref. [58]. (**C**) Schematic illustration of the pH-responsive simultaneous drug release and cellular uptake for synergistic tumor phototherapy. ECM: extracellular matrix. Adapted with permission from ref. [62]. (**D**) Schematic mechanism of the BDPmPh, BDPbiPh and BDPtriPh NPs applied in lower pH-activated PDT/PTT. Adapted with the open access figure from ref. [63].

Compared to chemotherapy, pH-triggered drug release is not frequently applied in synergistic phototherapy via PDT and PTT, probably due to the difficulty or unnecessity of conjugating PS to the nanocarriers. In one study, chitosan was used as a gatekeeper in response to the lower pH to prevent the premature release of loaded MB in iron oxide-based hybrid nanoassemblies (NAs) for low-power-assisted PDT/PTT [55]. The formed MB-NAs produced both hyperthermia and singlet oxygen under low-power NIR irradiation in vitro and in vivo, which effectively improved antitumor performance. In addition, Xu and colleagues developed a multi-stimulus-initiated release strategy including pH to realize fluorescence imaging-guided synergistic PDT/PTT of HER2-overexpressed breast cancer [56]. The multi-purpose nanoplatform was prepared by the modification of gold nanorods (GNRs) with HA-functioned pendant hydrazide and thiol groups via Au-S bonds, and subsequently grafting 5-aminolevulinic acid (ALA), Cy7.5 and anti-HER2 antibody onto HA moiety for PDT, fluorescence imaging and active targeting, respectively (Figure 3A). The intracellular release of ALA was activated by the acidic cleavage of hydrazone bonds. At the same time, the HA coating could be degraded by intracellular GSH and lysosomal enzyme HAase, further accelerating the release of ALA and Cy7.5. Upon fluorescence imaging-guided NIR irradiation, the dual-targeted and TME-responsive nanoplatform produced an optimal therapeutic efficacy with negligible adverse effects by combined PDT/PTT therapy.

### 3.2. Low pH-Enhanced Cellular Uptake

Compared to the modulated release of PS or photothermal agents, pH has been more frequently employed for enhanced cellular uptake due to the protonation of nanocarriers in synergistic phototherapy. For instance, Zhang and co-workers designed dual-responsive polypeptide nanoparticles for phototherapy [57]. The ICG-loaded NPs were composed of pyridine dithioethylamine-modified poly(L-lysine) (PLL_PDA_) and thiolated PLL (PLL_SH_). PEG and dimethylmaleic anhydride (DMMA) were decorated on the surface of PLL NPs (PLL-ICG/DPEG NPs) to increase the hydrophilicity. The stripped DMMA chains at acidic pH inverted the zeta potential of PLL NPs from negative to positive, consequently leading to high cell association and uptake. Eventually, the thermo-responsiveness and ROS generation of the loaded ICG in DMMA-modified PLL NPs induced improved cytotoxicity against cancer cells in vitro compared to succinic anhydride (SA)-modified PLL NPs without pH-responsiveness.

With a different design but a similar aim, Han’s team reported rationally designed pH-responsive polymeric micelles to realize PTT and photothermally triggered oxygen-independent combined PDT [58]. They synthesized a triblock copolymer, poly(ethylene glycol)-b-poly(ε-caprolactone)-b-poly(2-(piperidin-1-yl)ethyl methacrylate) (PEG-b-PCL-b-PPEMA) and encapsulated the PS cypate and singlet oxygen donor (diphenylanthracene endoperoxide, DPAE) via a self-assembly strategy to fabricate the micellar delivery system (C/O@N-Micelle) (Figure 3B). Such micelles displayed enhanced tumor accumulation and improved cellular uptake (2.1 times) as the pH value dropped from 7.4 in blood circulation to 6.8 in tumor tissues, as a result of the protonation of PPEMA chains and the resultant surface charge reversal. More interestingly, the micelles were able to yield potent hyperthermia for PTT by cypate under 808 nm NIR irradiation, which simultaneously activated the thermal cycloreversion of DPAE to produce adequate singlet oxygen for PDT without the participation of oxygen. Very recently, Gao’s team reported a pH-responsive nanoplatform composed of Ce6-loaded PLGA NPs which was decorated with PDA before the modification with PAHDMMA for long blood circulation [59]. The NPs displayed enhanced tumor accumulation and improved cellular uptake due to the charge reversal of DMMA in response to the pH decrease in TME. The irradiation with dual-wavelength laser at 660 nm and 808 nm triggered the photothermal effect of PDA and the ROS generation by Ce6, respectively for combined tumor phototherapy.

Based on similar principles, Xie and his team successfully engineered a pH-responsive nanocluster (NC) composed of indocyanine green (ICG), Fe_3_O_4_, and palmitoyl ascorbic acid (PA) with a pH-triggered surface charge reversal polymer PEG-b-PAEMA-DMA for synergistic enhancement of phototherapy, where ICG performed as both PS and photothermal agent [60]. Under laser irradiation at 808 nm, NCs not only produced significant hyperthermia for PTT but also generated ^1^O_2_ and H_2_O_2_ to accelerate the PDT, thereby enhancing the efficacy of combination therapy. More importantly, NCs significantly improved intracellular uptake at low pH via the charge reversal, and thus induced higher tumor suppression by the combined phototherapy.

More recently, Lin’s team designed a programmed stimuli-responsive poly(N-isopropylacrylamide)-carbon dot (PNIPAM-CD) hybrid nanogels consisting of hydrophilic/hydrophobic convertible PNIPAM, pH-responsive N-methylallylamine (MAA), and redox-sensitive N,N′-bis(acryloyl)cystamine (BAC) [28]. Under 660 nm laser irradiation, CD could generate both hyperthermia and ROS for PTT and PDT, respectively. Furthermore, cellular uptake could be boosted due to the pH-responsive charge inversion and temperature-dependent hydrophilic/hydrophobic conversion properties of PNIPAM-CD.

### 3.3. pH-Responsive Drug Release and Cellular Uptake

Beyond the single application in response to acidic TME, there have also been studies taking advantage of lower pH for both triggered drug release and enhanced cellular uptake. A new type of design named pH-driven insertion of nanocarriers into cell membranes is reported by Yu and co-workers who modified Ce6-loaded hollow gold nanospheres (HAuNS) with pH-responsive insertion peptide (pHLIP) [61]. Interestingly, pHLIP kept the α-helix conformation in physiological pH conditions but transformed into the inserted state at pH 6.2, which was close to the acidic TME. This pH-responsiveness allowed for the transmembrane ability of nanocarriers for boosted cellular uptake. Afterward, HAuNS showed strong photothermal coupling properties under light irradiation, which generated heat for PTT and provoked the release of Ce6 and pHLIP from the surface of HAuNS. In short, this study presented a step-by-step pH-responsive tumor phototherapy.

Another more interesting example is presented by Peng et al., who modified two-dimensional molybdenum disulfide (MoS_2_) nanosheets with a pH-responsive peptide (LA-K_11_ (DMA)), and then loaded the positively charged photosensitizer toluidine blue O (TBO) onto MoS_2_ through physical absorption (designed as MKT) (Figure 3C) [62]. At pH 7.4, the fluorescence and photo-induced ROS generation of TBO were severely quenched by two-dimensional MoS_2_ owing to an instantaneous Förster resonance energy transfer [64]. At lower pH in the TME, the lysyl succinyl amides of LA-K_11_(DMA) were hydrolyzed, thereby exposing the positively charged amino groups [65]. The charge-reversed nanoplatform displayed a higher affinity toward negatively charged cell membranes for cellular uptake. Meanwhile, the positively charged peptide performed as a “spy”, which could reduce interactions between the absorbed TBO and the two-dimensional MoS_2_, eventually leading to the release of TBO. This dual-function of pH responsiveness endowed the nanoplatform with an effective therapeutic effect via TBO-based PDT and MoS_2_-based PTT under light irradiation.

### 3.4. Low pH-Activated Phototherapy

In addition to pH-triggered cargo release and charge reversal-enhanced cellular internalization, recent years have also witnessed a lower pH-activated combination of PDT and PTT with nanocarriers. In a leading study, Zou et al. designed three phenyl-based boron dipyrromethene (BODIPY) compounds conjugated with different numbers of diethylaminophenyl groups onto the BODIPY core and fabricated NPs with them via a nanoprecipitation method [63]. By varying the conjugation degree (Figure 3D), the NIR absorbance of these compounds (BDPmPh, BDPbiPh, and BDPtriPh) could be tailored to control the intratumoral penetration depth. It is assumed that the pH responsiveness of the BDPmPh, BDPbiPh and BDPtriPh NPs resulted from the photoinduced electron transfer (PET) mechanism. Under neutral conditions, the HOMO level of the -NEt_2_ group stayed between the HOMO and LUMO levels of the PS molecule. Therefore, the excited –NEt_2_ groups conveyed an electron to the HOMO of the PS, while the excited electron of the PS was inclined to be shunted to the HOMO of the –NEt_2_ group instead of experiencing intersystem crossing or radioactive/vibrational relaxation. In contrast, under acidic conditions, the H^+^ attached –NEt_2_ group due to protonation made its HOMO lower than that of the PS, which would activate the photothermal and photodynamic activity of the PS.

In summary, lower pH in TME is frequently utilized for controlled drug release and improved cellular uptake in synergistic phototherapies owing to the protonation of the chargeable moieties in the nanocarriers, while both aims could also be realized at the same time when additional designs (e.g., “spy” molecules) are introduced into the nanomedicines. In addition, lower pH could also serve as the trigger of phototherapy to precisely control the tumor treatment. These studies greatly widen the applications of pH-responsive nanomedicines in tumor phototherapy with improvements in drug delivery to tumor cells, drug release, and controllable treatment activation.

## 4. pH and H_2_O_2_ Dual-Responsive Phototherapy

In synergistic phototherapy, the therapeutic effects are sometimes limited in single stimulus-responsive drug delivery systems (DDSs) due to the complexity of the tumor. Thus, it is necessary and beneficial to develop multiple stimuli-sensitive systems to further improve anti-cancer efficiency. Among the multimodal responsive nanocarriers, the dual-responsiveness to lower pH and enriched H_2_O_2_ is most widely employed in phototherapy (Table 3).

### 4.1. Enriched H_2_O_2_-Powered Hypoxia Relief via MnO_2_ Nanomaterials

**Figure 4:**
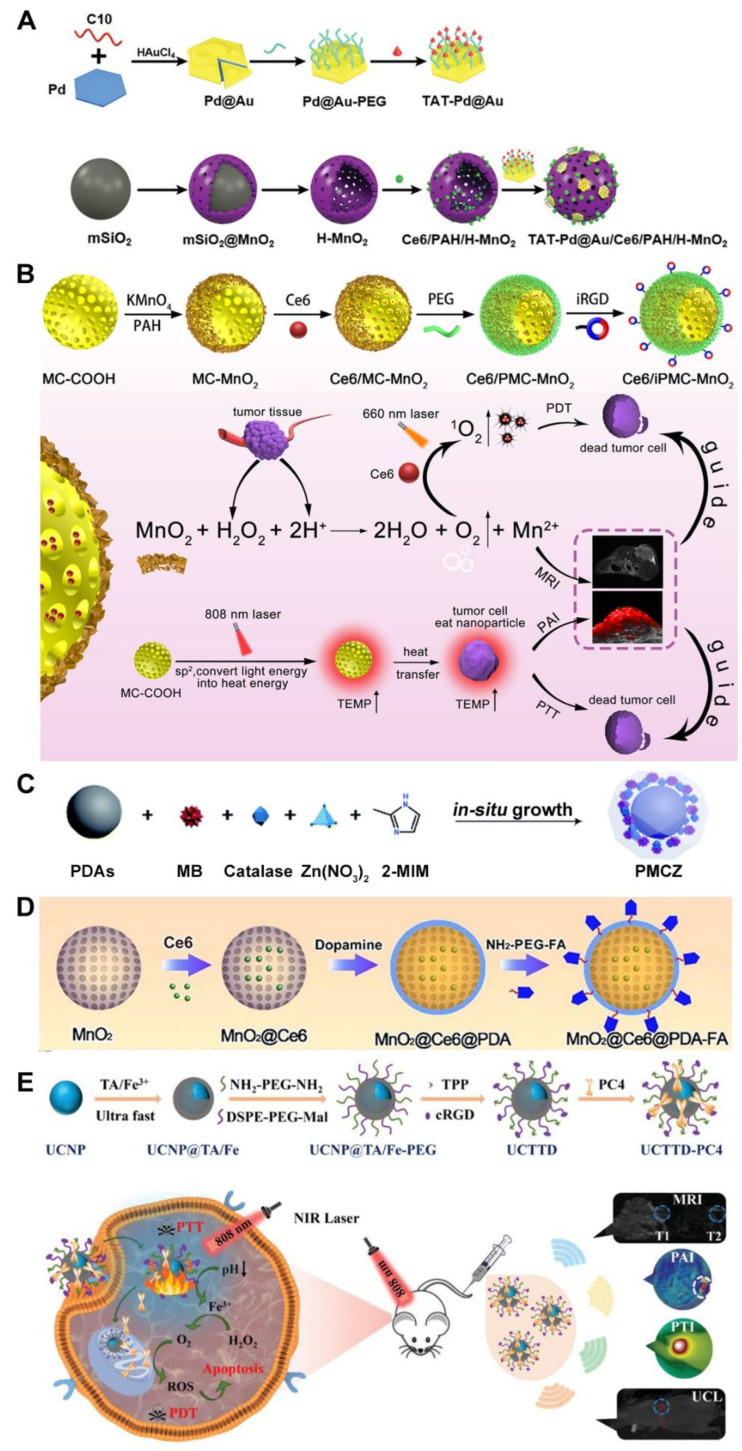
H_2_O_2_ and pH dual-responsive nanoplatforms for synergistic phototherapy with hypoxia relief and additional benefits including controlled drug release. (**A**) Schematic illustration of the construction of MnO_2_-based H_2_O_2_ and pH dual-responsive NPs for hypoxia relief in nucleus-targeted PTT and hypoxia-relieved PDT. Adapted with permission from ref. [66]. (**B**) Schematic illustration of the facile preparation of H_2_O_2_ and pH dual-responsive Ce6/iPMC-MnO_2_ and their application in dual-modal imaging-guided PDT and PTT. Adapted with permission from ref. [38]. (**C**) Schematic illustration of the fabrication of the intelligent ZIF-8-gated PDA nanohybrids for hypoxia relief with CAT in the combined PDT and PTT. Adapted with the open access figure from ref. [70]. (**D**) Schematic illustration of the fabrication of tumor-targeting NPs with dual-responsiveness for hypoxia relief and pH-triggered PS release. Adapted with permission from ref. [71]. (**E**) Schematic diagrams of the synthesis and application of UCTTD-PC4 nanoplatform for tumor-targeted PTT/PDT for hypoxic pancreatic cancer triggered by TME and guided by multimodal imaging. Adapted with permission from ref. [74].

As mentioned above, MnO_2_ can also relieve tumor hypoxia by triggering the decomposition of endogenous H_2_O_2_ in acidic TME and promote the therapeutic efficacies of combined PDT and PTT in addition to providing Mn^2+^ ions for magnetic resonance imaging (MRI) [78]. For example, Ce6-loaded hollow mesoporous MnO_2_ NPs were used as the core and decorated with bimetallic Pd@Au nanoplates and cell nucleus-target transactivator of transcription (TAT) peptides for synergistic tumor therapy (Figure 4A) [66]. The Pd@Au nanoplates were synthesized from small Pd as seeds and DNA-induced morphological control strategy [79], which displayed a photothermal conversion efficiency up to 57%, thus overmatching other previously reported photothermal counterparts activated in the NIR-II region including Cu_9_S_5_ NPs (37%) [80], Cu_3_BiS_3_ nanorods (41%) [81], and Au/Cu_2−x_S nanocrystals (43%) [78]. The MnO_2_ nanocarrier could be rapidly decomposed by reacting with H_2_O_2_ at lower pH, generating O_2_ to overcome tumor hypoxia to improve the effectiveness of Ce6-mediated PDT, whereby the released small size Pd@Au nanoplates further targeted to the nucleus for NIR-II PTT. A similar strategy was also utilized in the intelligent nanotheranostics based on Au/Ag-MnO_2_ hollow nanospheres (AAM HNSs) [67], mesoporous carbon-manganese nanocomposite (MC-MnO_2_) (Figure 4B) [38] and a core-shell-shell multifunctional AuNRs (Au nanorods) @MnO_2_@SiO_2_ NPs [68]. Either Au/Ag alloy or MC in the studies revealed a remarkable photothermal conversion capability for PTT, which was assisted by the MnO_2_-enhanced PDT. More interestingly, dully assisted ICG-mediated PDT by oxygen generation and AuNRs-mediated PTT conferred excellent antitumor effects even with only one injection of NPs and NIR irradiation.

Besides, MnO_2_ nanomaterials have also been collaborating with non-traditional metallic single photosensitive agents for simultaneous PDT and PTT, such as Cu_2–x_S-coated MnO_2_ NPs (MnO_2_/Cu_2–*x*_S) [76]. The Cu_2–*x*_S NPs conducted PTT due to the distinct SPR band at the NIR region [77] and concomitantly produced ROS for parallel PDT. Importantly, the co-loaded siRNA targeting shock protein (HSP) 70 was able to block the heat-shock response, which concurrently boosted the efficacies of PTT for better therapeutic outcomes.

Another example worthy to mention reported an intelligent Bi/MnPcE_4_ nanocomposite for trimodal imaging (FL/CT/MRI)-guided, oxygenation-enhanced PDT and PTT [69]. Interestingly, these nanocomposites didn’t require the additional loading of PSs or photothermal agents. The nanocomposite not only exhibited high photothermal conversion efficiency (~34%) under 808 nm laser irradiation but also catalyzed H_2_O_2_ to boost the O_2_ level in the TME for enhanced PDT performance. In addition, Bi allowed the use of these nanocomposites in CT imaging, and Mn enabled them for MRI, all of which suggested a multimodal imaging capacity of the nanocomposites in the tumor.

### 4.2. Enriched H_2_O_2_-Powered Hypoxia Relief and pH-Responsive Drug Release

As a trigger responsive to enriched H_2_O_2_ for hypoxia relief, CAT also plays an important role together with low pH in dual-stimuli responsive synergistic phototherapy. For instance, Feng et al. synthesized multifunctional PDAs-MB-CAT-ZIF-8 (PMCZ) nanoparticles (Figure 4C) [70]. In an acidic TME, the metal-chelating ability of the catechol group of PDA and the superficial metal ions of zeolite imidazole salt skeleton 8 (ZIF-8) was weakened, allowing for the release of MB and CAT and making the ZIF-8 shell serving as a smart gatekeeper for drug release. CAT catalyzed the decomposition of H_2_O_2_ to produce O_2_ for enhancing MB-induced PDT. Meanwhile, PDAs generated heat under NIR light to induce the thermal ablation of cancerous cells to assist PDT.

In addition, the controlled PS release and oxygen replenishment have been simultaneously achieved via MnO_2_ nanomaterials and pH-responsive entities. Zeng and colleagues developed a multifunctional nanoplatform formed by hollow mesoporous MnO_2_ NPs loaded with Ce6 and further coated with folic acid-functionalized PDA (MnO_2_@Ce6@PDA-FA NPs, MCPF NPs) (Figure 4D) [71]. MCPF NPs were able to avoid the premature release of Ce6 in the blood circulation due to the appreciable stability of the PDA shell at pH 7.4. When the NPs accumulated in the tumor (pH 6.8), the PDA shell was degraded, acting as a gatekeeper to release Ce6 in the acidic environment and expose MnO_2_ for improved PDT, while the NPs underwent photothermal conversion for PTT, which further accelerated the release of Ce6 and O_2_ generation to ablate the tumor. In a similar way, Zhang et al. reported PDA-coated mesoporous silica nanoparticle (MSN) core encapsulating Ce6 and CuS NPs) as the photodynamic and photothermal agents, respectively [72].

Very recently, Li and his team constructed an environmentally responsive biomimetic MOF-based drug delivery system with catalase-like activity (FA-EM@MnO_2_/ZIF-8/ICG) for the synergistic phototherapy of tumors [73]. The ZIF-8/ICG nanocore was easy to decompose in the acidic for controllable ICG release. It was coated with MnO_2_ for self-supply of O_2_ in the presence of H_2_O_2_ in TME to improve the PDT efficacy which efficiently suppressed tumor growth together with PTT, while the wrapping of folate-functionalized erythrocyte membrane (EM) provided longer systemic circulation and active targeting.

In addition, Wang and his team recently prepared a smart nanoplatform with dual TME responsiveness using a single NIR laser to induce synergistic PDT/PTT which additionally aimed for tackling the light penetration issue, one of the inherent drawbacks of PDT [74]. Lanthanide-doped upconversion nanoparticles (UCNPs) with narrow emission peaks, outstanding photostability, and high tissue penetration depth could convert NIR light to visible or UV emission. Tannic acid (TA), an organic ligand, that could form stable complexes with metal ions such as Fe^3+^ at neutral pH, was employed to generate TA/Fe^3+^ nanofilms onto the UCNPs (Figure 4E). Interestingly, they tended to dissociate at acidic TME and release the PS and Fe^3+^. The former could realize a synergistic PTT/PDT effect via the absorption of emissions generated by UCNPs irradiated with a single NIR laser at 808 nm, while the latter could catalyze endogenous H_2_O_2_ into O_2_ to alleviate tumor hypoxia and enhance the PDT efficacy. In short, this study delivers a simple and smart manner to create a collaborative PTT/PDT nanoplatform by a single NIR laser irradiation that could achieve hypoxia relief and controlled PS release via the stepwise responsiveness to low pH and enriched H_2_O_2_ in the TME.

### 4.3. Enriched H_2_O_2_-Powered Hypoxia Relief and pH-Responsive Enhanced Intratumoral Drug Penetration

Finally, MnO_2_ degradation in response to acidic and H_2_O_2_-enriched TME also provides additional benefits of deeper intratumoral diffusion. To realize this purpose, Liu and colleagues developed a new honeycomb MnO_2_-based nanoplatform to achieve excellent phototherapy [75]. In detail, IR780 and BSA were adsorbed onto honeycomb MnO_2_ (HMIB NPs). Once the HMIB NPs were accumulated in tumor tissue, MnO_2_ was gradually degraded and supplied O_2_ in response to H_2_O_2_ and H^+^ in TME, which produced smaller MnO_2_ NPs and the released IR780-BSA complex for deeper intratumoral penetration. By combining the photothermal and photodynamic effects of IR780 with TME responsive size-tunability and O_2_ self-supply of honeycomb MnO_2_, HMIB NPs demonstrated great promise for synergistic PDT/PTT.

In short, dual responsiveness to acidic and H_2_O_2_-rich TME has played an important role in improving synergistic phototherapy. Taking advantage of the catalysis and decomposition in such conditions, MnO_2_ has been extensively applied in the dual-responsive nanomedicines for boosted phototherapy via hypoxia relief and decreased size for better tumor penetration of nanomedicines. In addition, hypoxia relief could also be realized via catalysis by CAT and other metallic nanomaterials which could conduct PDT and PTT at the same time. Low pH-triggered drug release has also been additionally achieved by the gatekeeping materials in the nanoplatforms such as ZIF-8, PDA and TA/Fe^3+^ nanofilms which are vulnerable to tumor acidity. In one word, the dual responsiveness to lower pH and H_2_O_2_ brings the benefits of both aspects in boosting the efficacy of phototherapy.

## 5. TME-Responsive PDT/PTT/Chemotherapy

As mentioned above, dual-modal synergistic phototherapy including PDT and PTT has become increasingly popular because of their unique advantages. Compared to dual-modal therapy, although still in a nascent stage, tri-modal synergistic therapy can completely inhibit tumor progression in certain cases owing to multiple mechanisms of cell-killing effect without causing obvious side effects or damage to normal tissues [13]. In this section, we will discuss the most commonly employed tri-modal tumor therapy, the combination of PDT/PTT/chemotherapy due to the fierce applications of chemotherapy in both fundamental research and clinical practices, with particular attention to nanocarriers responsive to TME (Table 4).

### 5.1. TME-Responsive Hypoxia Relief and Drug Release for Enhanced PDT/PTT/Chemotherapy

**Figure 5:**
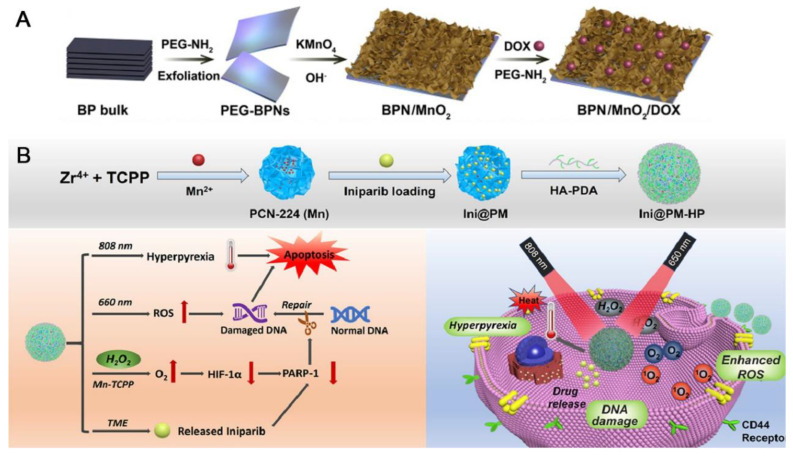
TME-responsive hypoxia relief in tri-modal synergistic PDT/PTT/chemotherapy. (**A**) Schematic illustration of the synthesis of multifunctional BPN/MnO_2_/DOX nanocomposites and the systemic delivery of BPN/MnO_2_/DOX as a versatile theranostic platform for MRI-guided multifunctional therapy. Adapted with permission from ref. [82]. (**B**) Schematic illustration of preparing Ini@PM-HP NPs and their applications in combinational tumor treatment. Adapted with permission from ref. [84].

Given the TME features, scientists developed mesoporous nanocarriers (e.g., the hydrangea-structured MnO_2_ NPs and mesoporous PDA) for the effective loading of PSs, photothermal agents and chemotherapeutic drugs [83,92], which could not only catalyze the conversion of endogenous H_2_O_2_ to O_2_ for tumor hypoxia relief and enhancement of ROS generation but also absorb light to generate heat, realizing the synergistic treatment of PDT/PTT/chemotherapy. Responsive to multiple TME stimuli, the hydrangea-structured MnO_2_ NPs were employed as the carrier of doxorubicin (DOX) and aza-BODIPY, which revealed rapid degradation in the presence of rich H_2_O_2_ and low pH, and accordingly enabled ^1^O_2_ generation and DOX release in the tumor to achieve a collaboration of oxygenation-enhanced PDT, PTT and chemotherapy [83]. In addition, a class of compact MnO_2_-laden black phosphorus (BPN/MnO_2_) nanostructure was fabricated by Wang’s team to load DOX for synergistic PDT-PTT-chemotherapy [82] (Figure 5A). The resultant BPN/MnO_2_ nanocarrier realized enhanced phototherapy due to the presence of MnO_2_ which performed not only as an effective hypoxia ameliorator and MRI contrast via the decomposition into Mn^2+^ but also as a photothermal-enhancing reagent. The nanocomposite also exhibited a smart drug release behavior in response to endogenous TME stimuli (pH, H_2_O_2_ and GSH) and exogenous photoirradiation in tumor lesions. Eventually, compared to traditional remedies, this self-driven intelligent theranostic nanoplatform demonstrated a remarkably enhanced therapeutic effectiveness with substantially reduced side effects.

More recently, Dong’s team constructed a biodegradable oxygen-producing nanoplatform to achieve coordinated PDT/PTT/chemotherapy [84]. The nanoplatform consisted of a zirconium-based MOF with the PS tetra(4-carboxyphenyl)porphyrin as a ligand for Mn chelation (Mn-TCPP), a polyadenosine diphosphate ribose polymerase (PARP) inhibitor (Iniparib), and PDA-modified hyaluronic acid (HA-PDA) for photothermal conversion and active targeting (named Ini@PM-HP) (Figure 5B). Notably, PDT enhancement can be achieved not only through the in situ generation of O_2_ by the reaction of Mn-TCPP with endogenous H_2_O_2_ to alleviate hypoxic TME, but also by driving high photothermal conversion of PDA under 808 nm laser irradiation to achieve PTT. In addition, iniparib could be released in the acidic tumor microenvironment owing to MOF decomposition, thereby dysregulating DNA damage repair and promoting apoptosis. Overall, this nanoplatform achieved efficient co-delivery and the combination of three therapies which realized effective inhibition of tumor growth, providing a promising strategy to overcome tumor hypoxia and achieve controllable drug release.

### 5.2. TME-Responsive Drug Release for Enhanced PDT/PTT/Chemotherapy

Besides the oxygen supply taking advantage of abundant H_2_O_2_ in the TME for boosted PDT in the tri-modal PDT/PTT/chemotherapy, the TME stimulus was also used to trigger the release of chemotherapeutic drugs and/or PSs by lower pH which could induce drug protonation [93], NP dissociation [87] and the breaking of the pH-sensitive bond [94] and other TME features such as enriched GSH.

Via the protonation strategy, Wang and co-workers designed micelles self-assembled from a pH-responsive diblock copolymer, a photosensitizer, and a polymeric prodrug of DOX for multimodal tumor imaging and combinational treatment of drug-resistant tumor [85]. Ce6 was chelated with the MRI reagent gadolinium(III) (Gd^3+^), upon cellular endocytosis, the micelles were quickly disintegrated in the early endosome via protonation and produced strong fluorescence and T_1_-weighted MR signals for imaging. The disassembly of micelles also allowed the generation of notable ROS for PDT, the temperature elevation for PTT and intratumoral penetration of the chemotherapeutics, where DOX was released from the prodrug via degradation of the GFLG spacer. Similarly, Qu’s team recently reported a novel sandwich nanostructure called Bi_2_Se_3_/MoSe_2_/Bi_2_Se_3_ (Bi-M-3) [86]. The z-scheme mechanism of charge transfer inside the nano heterostructure induced enhanced ROS generation owing to the efficient separation of photogenerated electron-hole pairs. Meanwhile, the nanostructure also revealed an extraordinary photothermal conversion efficiency approaching 60%, which in turn, boosted the transfer of photo-generated electrons that further promoted ROS production. More interestingly, the Bi-M-3 allowed acidity and photothermal effect-induced drug release profile of DOX for chemotherapy due to the dampened electrostatic interaction and π-π stacking, respectively, between DOX and B-M-3.

**Figure 6:**
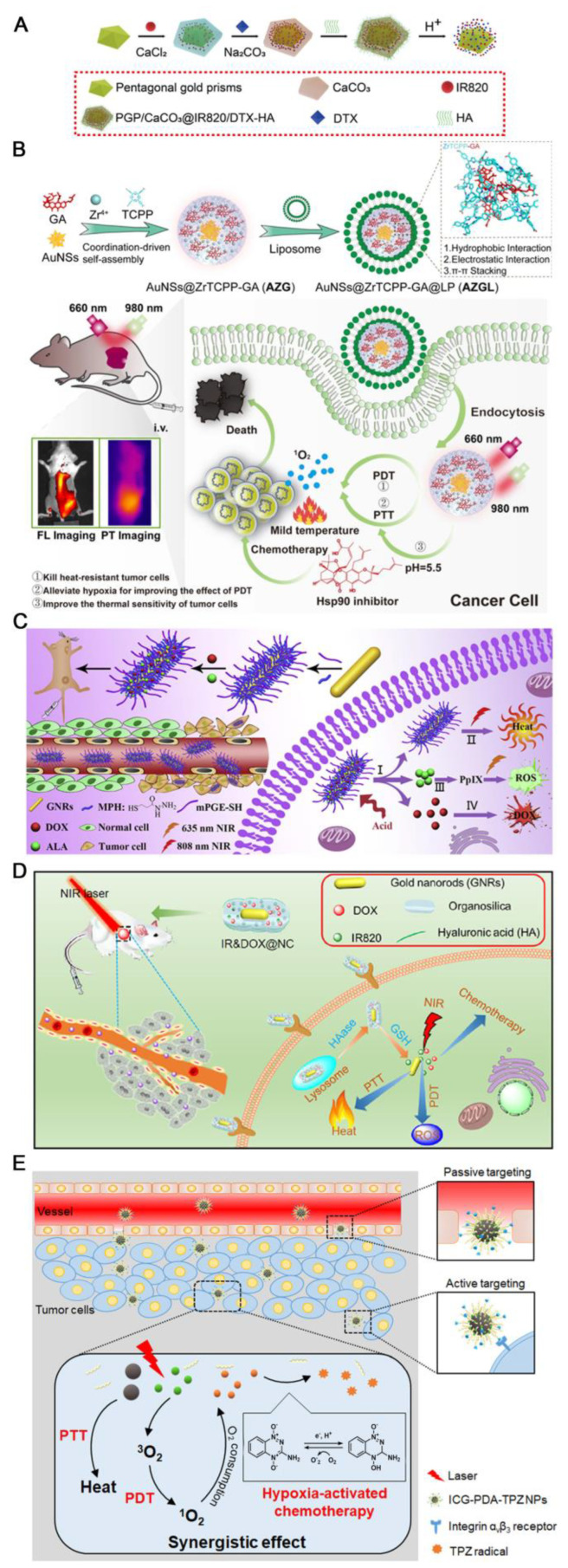
TME-responsive drug release including low pH/GSH/enzyme-induced protonation, NP self-immolation, bond cleavage and hypoxia-activated chemotherapy in tri-modal synergistic PDT/PTT/chemotherapy. (**A**) Schematic illustration of preparing PGP/CaCO_3_@IR820/DTX-HA nanoprobes. Adapted with permission from ref. [87]. (**B**) Schematic illustration of AZGL nanocomposite preparation and its application in breast cancer treatment. Adapted with the open access figure from ref. [88]. (**C**) Schematic illustration of the preparation process for GNRs-MPH^-ALA/DOX^-PEG and their application in tri-modal PDT/PTT/chemotherapy of breast cancer. (I) Simultaneous release of ALA and DOX provoked by acidic TME. (II) Hyperthermia generated from the GNRs-MPH-PEG for PTT. (III) ALA was metabolized to PpIX for PDT. (IV) DOX release for chemotherapy. Adapted with permission from ref. [89]. (**D**) Schematic illustration of IR&DOX@NC fabrication and intratumor process for multi-modal tumor ablation. Adapted with permission from ref. [90]. (**E**) Schematic illustration for the preparation of ICG-PDA-TPZ NPs and their combined PDT/PTT/Hypoxia-activated chemotherapy. Adapted with the open access figure from ref. [36].

Besides, Tan and colleagues developed a pH-initiated self-immolation strategy to realize controlled drug release and multimodal imaging-guided synergistic therapy of castration-resistant prostate cancer (CRPC) [87]. The pH-activated nanoprobes were prepared by modifying pentagonal gold prisms (PGPs) with CaCO_3_ shell loaded with IR820 and the chemotherapeutic docetaxel (DTX) (PGP/CaCO_3_@IR820/DTX), which were then decorated with hyaluronic acid (HA) for active targeting (Figure 6A). The pH-responsive decomposition of CaCO_3_ was capable of releasing IR820 and DTX for phototherapy and chemotherapy, respectively, which achieved optimal therapeutic efficacy with negligible adverse effects.

Not long ago, Chen’s team prepared a multifunctional, pH-responsive nanoplatform with a core-shell structure for the effective synergistic treatment of breast cancer [88]. Gold nanostars (AuNS) were used to direct the growth of MOF composed of Zr^4+^ and TCPP, while gambogic acid (GA) was encapsulated by a simple coordination reaction, followed by the coating of polyethylene glycolized liposomes (LP) on the surface of the nanometallic organic backbone (NMOF) to enhance stability and biocompatibility (Figure 6B). The resultant AuNS@ZrTCPP-GA@LP (AZGL) nanocomposites integrated AuNS-mediated mild PTT, TCPP-mediated PDT, and GA-mediated chemotherapy for synergistic treatments of breast cancer. The NMOF of ZrTCPP in AZGL was slowly degraded in the weakly acidic TME, releasing AuNS, Zr^4+^, GA and TCPP, which could effectively produce ROS under 660 nm laser irradiation. Besides, the heat generated by the photothermal effect of AZGL not only killed cancer cells but also alleviated the hypoxia of solid tumors, further enhancing the PDT effect. In addition, in addition to the chemotherapy effect, GA blocked HSP90 which was overexpressed by heat stress, making the cells sensitive to PTT. Consequently, such synergistic tri-modal therapy resulted in a strong antitumor effect against breast cancer.

Taking advantage of pH-sensitive bond cleavage, gold nano rods (GNRs) were decorated with mercaptopropionylhydrazide (MPH) and mPEG-SH via Au-thiol linkage, and subsequently conjugated with DOX and pro-PS 5-aminolevulinic acid (ALA) through acid-liable hydrazone bonds between drugs and MPH molecules [89]. The resultant GNRs-MPH^-ALA/DOX^-PEG NPs displayed pH-responsive release behaviors of DOX and ALA in the tumor site (Figure 6C). The former led to significantly enhanced blood circulation of NPs and consequently boosted tumor accumulation of NPs up to 3.3%, while the later enabled chemotherapy and the metabolism of ALA into protoporphyrin IX (PpIX), a PS to yield enough ROS for PDT under NIR irradiation. Meanwhile, GNRs could efficiently induce hyperthermia for PTT. Compared to single chemotherapy and dual-modal chemotherapy/PDT or chemotherapy/PTT, the tri-modal chemotherapy/PDT/PTT could more efficiently kill MCF-7 cells via a superadditive antitumor effect.

Employing the enriched GSH and enzyme, Cheng and co-workers designed a dual-responsive nanohybrid for multimodal tumor treatment [90]. The gold nanorods were coated with mesoporous silica, which was loaded with DOX and IR820 as both the PS and photothermal agent and modified with HA (IR&DOX@NC) for tumor targeting and improved biocompatibility. The prepared nanohybrids exhibited enhanced intracellular release of DOX and IR820 triggered by the degradation of HA and organosilica in responsive to HAase and GSH (Figure 6D). Under 808 nm light irradiation, IR&DOX@NC triggered not only the generation of ROS but also remarkable photothermal efficacy originating from gold nanorods, achieving a combinatorial photodynamic, photothermal and chemotherapy for highly efficient antitumor outcome in vitro and in vivo.

Very recently, Luo’s team reported a traceable, ROS-responsive nanosystem for integrated chemotherapy/PTT/PDT, which was self-assembled by an IR825-bonded N-isopropylacrylamide modified lignin (MND-IR) and a natural plant antitoxin compound resveratrol (RESV) (MND-IR@RESV) [91]. The MNDIR@RESV micelles could release RESV at the tumor site in response to NIR irradiation, which induced the photothermal conversion of IR825 and the hydrophilic to hydrophobic transition of PNIPAM chains for the disintegration of the micelles into flocculent insoluble substances. MND-IR@RESV also responded significantly to ROS, which promoted the deep drug release. The high photothermal conversion efficiency and photothermal stability also allowed spatiotemporal targeting of drugs and the efficient tumor eradication ability of combination therapy together with RESV-mediated chemotherapy.

### 5.3. TME-Responsive Drug Release and Treatment Activation for Enhanced PDT/PTT/Chemotherapy

In tri-modal tumor therapy, recent years have also seen the application of NPs sensitive to TME stimuli for both drug release and chemotherapy activation. For instance, Huang et al. prepared acid-responsive tirapazamine (TPZ), ICG and PDA co-loaded CaCO_3_ nanoplatform modified with D-α-tocopheryl polyethylene glycol 1000 succinate (TPGS) and Arg-Gly-Asp (RGD) peptide for active targeting (ICG-PDA-TPZ NPs) [36]. In the acidic tumor microenvironment, the degradation of CaCO_3_ was conducive to the release of TPZ and ICG, while ICG-induced PDT aggravated further hypoxia to provoke the activity of TPZ for chemotherapy (Figure 6E). Meanwhile, as a photothermal conversion agent with excellent biocompatibility and biodegradability, PDA revealed evident photothermal effects in vivo together with ICG. Compared to other groups of single and dual-modal therapies, the combination of PDT/PTT/chemotherapy exerted by ICG-PDA-TPZ NPs exhibited the best antitumor efficacy.

In one word, in accordance with researchers’ intensions, the combination of PDT with PTT or chemotherapy and tri-modal therapies based on them have been extensively explored via nanoplatforms responsive to single or multiple TME stimuli, and have demonstrated further improved therapeutic effects, with the achievement of benefits including self-supplied oxygen, single or multiple TME-responsive drug release/activation as well as improved tumor accumulation and uptake. Given this, tri-modal synergistic tumor therapy via PDT, PTT and chemotherapy is expected to undergo further investigations in pre-clinical and potential clinical studies and they are believed to pave a substantial new avenue for cancer treatment in the future.

## 6. Conclusions and Perspectives

In summary, PDT has displayed intriguing potential in combating various malignant tumors. Although a few PSs have been approved for clinical applications, the full potential of PDT as a first-line treatment has not been fully substantiated owing to the TME conditions unamiable for the treatment such as hypoxia, upregulated antioxidants, as well as the weakness in intratumoral drug delivery and control of drug release. Consequently, it provides a strong scientific rational to combine PDT with other tumor therapies especially PTT and in certain cases together with chemotherapy. Herein, given the recent developments in nanomedicines, we present a comprehensive summary of nanomedicine design and the mechanisms of combined phototherapy and their combinations with chemotherapy for further boosted tumor ablation, focusing on those sophisticated nanocarriers composed of a broad spectrum of moieties with specific responsiveness to various TME stimuli to achieve additional benefits for bolstered tumor therapy, such as improved oxygen levels for ROS generation via H_2_O_2_ sacrifice, controlled drug release or therapy activation triggered via responsiveness to lower pH, enriched GSH and enzymes, as well as improved cellular uptake and enhanced intratrumoral penetration of nanomedicines.

Given the improvements and benefits mentioned above, it is suggested that nanomedicines bring tremendous potential to take advantage of TME features and consequently reinforce combined phototherapies. However, most of the nanoplatforms are still in the preclinical research stage, thereby further investigations are needed to overcome critical challenges before entering clinical applications. Firstly, the biocompatibility, immunogenicity, and pharmacokinetics of these nanocarriers need to be systemically evaluated. A great majority of the nanocarriers for combined phototherapy make use of a broad spectrum of organic and inorganic materials from modified natural polymers to synthetic polymer and polymer-drug conjugates, as well as mesoporous silica and particularly metallic nanomaterials, which have been partially and conditionally defined as “biocompatible”. Nevertheless, the in vivo degradation profiles of such nanocarriers, especially those inorganic materials with catalytic activity for hypoxia relief, and the long-term toxicity of themselves and the degraded products should also be carefully investigated. Secondly, the endowment of nanomedicines with smart TME responsiveness brings noticeable difficulties and complexities in nanocarrier fabrication (e.g., covalent conjugation and non-covalent coating), which will greatly hamper their scaled-up manufacturing for clinical translation. On the other side, it is worth noticing that liposomes and protein-based nanomedicine (e.g., serum albumin) loaded with chemotherapeutic drugs (e.g., paclitaxel and DOX), have been approved for clinical application around the world owing to their high biocompatibility, biosafety and flexibility of scaled-up manufacturing and the relatively facile preparation procedures exempted from complicated chemical synthesis (e.g., emulsification-solvent evaporation and self-assembly) [95]. Thus, we speculate a better and more reasonable development towards liposomes and albumin NPs with limited chemical synthesis in terms of formulating nanomedicines for combined phototherapy.

Besides the drawbacks of nanomedicines, there have also been a few critical obstacles to light-initiated phototherapy due to their inherent characteristics. Firstly, the efficacy of phototherapy is somehow frustrated by the limited penetration depth of light, although an NIR laser is employed in most cases. The penetration depth and delivery efficiency of light stand for two major obstacles of cancer phototherapy for deep tissue treatment due to the reflection and decay owing to the light-tissue (e.g., skin) interactions [96], which could be minimized with the increase of light wavelength [97]. Although NIR lasers with longer wavelengths have been extensively employed in phototherapy, they are generally fitted to superficial tumors since lasers within this wavelength range only possess a tissue penetration of around 5–7 mm [98]. To realize the delivery of light into deep-seated or large tumors, the light could be introduced into tumors with optical fibers equipped with a light delivery/dosimetry device, or via various strategies that could improve the light accessibility, such as light transducers (e.g., upconversion NPs and two-photon excited NPs), which can absorb light in the NIR region and emit in the visible region to activate PSs with the corresponding absorption [97]. Secondly, despite various strategies that could relieve tumor hypoxia via decomposing the enriched H_2_O_2_, the limited overall H_2_O_2_ level in TME largely impedes the efficacy of oxygen supply that could fully potentiate PDT and the resultant combined phototherapy [13]. Potential ways to solve this problem rely on the delivery of exogenous oxygen via nanomedicines (e.g., hemoglobin and perfluorocarbon) [99,100] and in situ oxygen generation from water in the tumors with catalytic substrates (e.g., CaO_2_) [101]. Moreover, type I PDT omitting the participation of molecular oxygen for ROS generation emerges as another powerful strategy to tackle the hypoxia limitation of phototherapy [102]. Proper design of novel PSs such as tetrapyrrolic and non-tetrapyrrolic ones (e.g., modified phthalocyanine and BODIPY) could effectively endow PDT with independence on oxygen level in the tumors. The third challenge of phototherapy resides in the precise quantification of dosage. The efficacy of ROS generation and photothermal conversion depends on a few parameters that are difficult to standardize, such as the physiochemical properties of the PS and photothermal agent (e.g., absorption coefficient and quantum yield), the local concentration of PS, photothermal agents and oxygen in the tumors, and the parameters of irradiation, particularly the fluence rate (W/cm^2^) and irradiation duration [97,103]. In addition, the decay of light intensity in vivo may also vary from case to case depending on the depth/size of tumors that may alter the light-tissue interactions.

The last critical challenge comes from complicated factors of the TME. For instance, the level of attenuated oxygen, decreased pH and other parameters such as the concentration of H_2_O_2_ and GSH may vary among different tumor types, and among different individuals with the same type of tumor, and may even evolve as the tumor develops in the same individual with the same tumor type. This renders the responsiveness of nanomedicines inaccurate or inadequate in controlling drug accumulation, release and activation for cell killing. In one word, with all the above-mentioned issues tackled, it is expected that TME-responsive nanomedicines will open up a new avenue for combined phototherapy and its collaboration with chemotherapy to facilitate their success in clinical cancer treatment.

## Figures and Tables

**Table 1:** Summary of phototherapies enhanced by hypoxia relief via catalytic H_2_O_2_ decomposition in the TME.

PS	Photothermal Agent	H_2_O_2_-Decomposition Entity	Function/Purpose	Tumor Model In Vitro	Tumor Model In Vivo	Ref.
MB	APMs	CAT	Controlled Release and hypoxia relief	PC-3 cells	/	[40]
Ce6	GNS	CAT	Hypoxia relief, in vivo CT imaging	HeLa and MCF-7 cells	BALB/c nude mice with HeLa tumors	[41]
Ce6	Pd@Pt	Pt	Hypoxia relief and tumor imaging	4T1 cells	Balb/c mice with 4T1 tumors	[42]
Ce6	PTAs	CAT	Hypoxia relief	HeLa cells	Balb/c mice with U14 tumors	[43]
Pt	Pd@Pt	Pt	Hypoxia relief	LM8 and L929 cells	Female Balb/c mice with LM8 tumors	[44]
ICG	GNSs	Pt	Hypoxia relief	4T1 cells	Mice with 4T1 tumors	[45]
ZnPc	PB	PB	Hypoxia relief	4T1 cells	Mice with 4T1 tumors	[46]
IR808-Br_2_	RuO_2_@BSA	RuO_2_@BSA	Hypoxia relief and NIRF imaging	4T1 cells	Mice with 4T1 tumors	[47]
ICG	Rh	Au@Rh	Hypoxia relief	MDA-MB-231 cells	Balb/c nude mice with MDA-MB-231 tumors	[48]
Ce6	MPDAand Rh NPs	Rh NPs	Hypoxia relief	4T1 cells	Female BALB/c mice with 4T1 tumors	[49]
BSA-IrO_2_	BSA-IrO_2_	BSA-IrO_2_	Hypoxia relief and low-temperature PTT	L929 cells	Mice with MDA-MB-231 tumors	[50]
Ce6	PDA and IrO_2_ NPs	IrO_2_	Hypoxia relief	HT29 cells	Balb/c nude mice with HT29 tumors	[51]

**Table 2:** Summary of phototherapies by nanomedicines responsive to lower pH in the TME.

PS	Photothermal Agent	pH-Responsive Entity	Function/Purpose	Tumor Model In Vitro	Tumor Model In Vivo	Reference
MB	IONPs	Chitosan.	Controlled drug release	HeLa, A549 and MCF-7 cells	BALB/c nude mice with A549 tumors	[55]
ALA	GNRs	hydrazone bonds	Controlled drug release	MCF-7 cells	Mice with MCF-7 tumors	[56]
ICG	ICG	DMMA	Enhanced cellular uptake	HeLa cells	/	[57]
DPAE	Cypate	C/O@N-Micelle	Enhanced tumor accumulation and improved cellular uptake	4T1 cells	BALB/c mice with 4T1 tumors	[58]
Ce6	PDA	PAH-DMMA	Enhanced cellular uptake	MCF-7 cells	/	[59]
ICG	ICG	PEG-b-PAEMA-DMA	Enhanced cellular uptake	HNE-1 cells	BALB/c mice with HNE-1 tumors	[60]
CDs	CDs	MAA	Enhanced cellular uptake	4T1 and MCF-7 cells	Nude mice with 4T1 tumors	[28]
Ce6	HAuNS	pHLIP	Drug release and cellular uptake	Hela cells	Nude mice with Hela tumors	[61]
TBO	MoS_2_	LA-K_11_ (DMA)	Drug release and cellular uptake	SCC-7 cells	BALB/c nude mice with SCC-7 tumors	[62]
BDPmPh, BDPbiPh and BDPtriPh NPs	BDPmPh, BDPbiPh and BDPtriPh NPs	Diethylamino groups	Low pH-activated phototherapy	HeLa cells	Nude mice with HeLa tumors	[63]

**Table 3:** Summary of phototherapies responsive to both lower pH and enriched H_2_O_2_ in the TME.

PS	Photothermal Agent	pH-Responsive Entity	H_2_O_2_-Responsive Entity	Function/Purpose	Tumor Model In Vitro	Tumor Model In Vivo	Reference
Ce6	Pd@Au nanoplates	MnO_2_	MnO_2_	Hypoxia relief	MCF-7 cells	BALB/c mice with MCF-7 tumors	[66]
Ce6	MC	MnO_2_	MnO_2_	Hypoxia relief	4T1 cells	BALB/c mice with 4T1 tumors	[38]
Ce6	Au/Ag alloy	MnO_2_	MnO_2_	Hypoxia relief	HeLa cells	Mice with HeLa tumors	[67]
ICG	AuNRs	MnO_2_	MnO_2_	Modulate the tumor microenvironment	MCF-7 cells	BALB/c mice with 4T1 tumors	[68]
MnPcE_4_	Bi	MnO_2_	Mn^2+^	Hypoxia relief	HeLa cells	Kunming mice with U14 tumors	[69]
MB	PDA	ZIF-8	CAT	Hypoxia relief and lowpH-triggered drug release	HeLa cells	Mice with HeLa tumors	[70]
Ce6	MnO2@Ce6@PDA-FA NPs	PDA	MnO_2_	Hypoxia relief and low pH-triggered drug release	MCF-7 and NP69 cells	BALB/c nude mice with MCF-7 tumors	[71]
Ce6	CuS	PDA	MnO_2_	Hypoxia relief and low pH-triggered drug release	4T1 cells	BALB/c mice with 4T1 tumors	[72]
ICG	ICG	ZIF-8	MnO_2_	Hypoxia relief and low pH-triggered drug release	4T1 cells	BALB/c mice with 4T1 tumors	[73]
PC4	TA/Fe^3+^ nanofilms	UCTTD	Fe^3+^	Hypoxia relief and low pH-triggered drug release	Capan-1 cells	BALB/c-nude mice with Capan-1 tumors	[74]
IR780	IR780	MnO_2_	MnO_2_	Hypoxia relief and enhanced intratumoral drug penetration	HepG2 and 3T3 cells	Nude mice with HepG2 tumors	[75]
/	Cu_2–x_S	MnO_2_	MnO_2_	Hypoxia relief	A549 and MCF-7 cells	Female BALB/c nude mice with B16 tumors	[76]
/	Au@Cu_2−x_S	Au@Cu_2−x_S/DOX-PEG	/	Drug delivery	MCF-7 cells	BALB/c nude mice with A549 tumors	[77]

**Table 4:** Summary of tri-modal synergistic PDT/PTT/chemotherapy by nanomedicines responsive to TME stimuli.

PS	Photothermal Agent	Chemotherapeutic Drug	TME Involved	Entities Responsive to TME	Purpose	Tumor Model In Vivo	Reference
BPNs	BPN/MnO_2_	DOX	High level of H_2_O_2_, low pH	MnO_2_	Oxygen generation for hypoxia relief and PDT enhancement, controlled drug release	Mice with HeLa tumors	[82]
aza-BODIPY	aza-BODIPY	DOX	High level of H_2_O_2_, low pH	MnO_2_	Hypoxia relief and drug release	/	[83]
PCN-224(Mn)	PDA	Iniparib	High level of H_2_O_2_	Mn-TCPP	Hypoxia relief, drug release and PDT enhancement	BALB/c nude mice with MDA-MB-231 tumors	[84]
Ce6	PDPC micelles	PDOX	Low pH	PEG-b-PDPA	Controlled drug release	Nude mice with MCF-7/ADR tumors	[85]
MoSe_2_	Bi_2_Se_3_/MoSe_2_	DOX	Low pH	Bi-M-3@PEG-Dox	Controlled drug release	Mice with U14 tumors	[86]
IR820	IR820	DTX	Low pH	CaCO_3_	Controlled drug release	PC-3 xenograft tumor-bearing nude mice	[87]
TCPP	AuNS	GA	Low pH	ZrTCPP	Controlled drug release	BALB/c mice with 4T1 tumors	[88]
PpIX	GNRs	DOX	Low pH	MPH	Controlled drug release	BALB/c nude mice with MCF-7 tumors	[89]
IR820	IR820	DOX	GSH and HAase	HA and organosilica	Controlled drug release	Nude mice with 4T1 tumors	[90]
IR825	IR825	RESV	ROS	MND-IR@RESV	Enhanced cellular uptake	Nude mice with U14 tumors	[91]
ICG	PDA	TPZ	Low pH	CaCO_3_	Controlled drug release	Mice with U87MG tumors	[36]
